# Comparison of Syndecan-1 Immunohistochemical Expression in Lobular and Ductal Breast Carcinoma with Nodal Metastases

**DOI:** 10.1155/2018/9432375

**Published:** 2018-07-29

**Authors:** Ivana Miše, Majda Vučić

**Affiliations:** ^1^Department for Clinical Cytology, University Hospital “Sestre Milosrdnice”, Zagreb, Croatia; ^2^Department of Pathology, University Hospital “Sestre Milosrdnice”, Zagreb, Croatia

## Abstract

Syndecan-1 (Sdc1) is a transmembrane heparan sulfate proteoglycan, an extracellular matrix receptor involved in intercellular communication, proliferation, angiogenesis, and metastasis. This study determined and compared Sdc1 expression in the tumor cells and stroma of 30 invasive lobular and 30 invasive ductal breast carcinomas (ILCs/IDCs), also in the axillary node metastases of ductal type, and correlated it with clinical and tumor parameters. Sdc1 was expressed in the epithelium of 90% carcinoma of both histological types. Also, it was most frequently expressed in their tumor stroma, but in ILC, stromal expression was negative in 40%. Sdc1 was expressed in 86.7% of the metastatic epithelium of IDC nodal metastases (in even 50% as high expression), while the nodal stroma was negative in 46.7%. Primary IDC showed a negative correlation between epithelial Sdc1 and progesterone receptors (PRs), whereas ILC showed a positive correlation between stromal Sdc1 and histological gradus. In the metastatic epithelium, Sdc1 was negatively correlated with a patient's age, estrogen receptors (ERs), and PRs in the primary tumors, while the stroma of metastases demonstrated a positive correlation with the focus number in primary tumors and a negative correlation with PRs in primary tumors. This research revealed identical overall epithelial Sdc1 expression in both breast carcinomas with no statistically significant difference in its stromal expression and confirmed the role of Sdc1 in the progression of both tumor types and in the development of ductal carcinoma's metastatic potential.

## 1. Introduction

Breast cancer is the most common and biologically very complex malignant tumor in women. Its incidence has been increasing lately, particularly in very young women, with often more aggressive clinical course and poor prognosis. There are numerous and intensive studies and discoveries of new molecular and genetic markers that could affect its development, growth, and potential treatment. One of them is the syndecan-1 (Sdc1), a transmembrane heparan sulfate proteoglycan (HSPG), the receptor for the extracellular matrix (ECM) and the organizer of the cell matrix adhesion which participates in tissue repair, metabolism, carcinogenesis, and development of the immune response; also, it integrates a variety of cellular signals and growth factor signals and modulates cell proliferation, migration, and angiogenesis [1–3]. As a coreceptor, Sdc1 binds, with heparan sulfate chains, to various growth factors and angiogenesis promoters by stimulating cell proliferation, and as an adhesion molecule, it enters into an interaction with ligands of ECM and cell surface. Sdc1 is the modulator of proteolytic activation and *in vivo* function of chemokines, which orchestrate leukocyte recruitment and tissue remodeling in inflammation and reparation [4]. It is predominantly expressed on/in epithelial cells, but it is also found in mesenchymal cells during their development and in the different stages of activation and differentiation of normal lymphoid cells—in centrocytes (but not in centroblasts), the mature plasma cells and the atypical plasma cells in the multiple myeloma, where it has been often investigated [5]. There are also indications that Sdc1 mediates the adhesion of the mesenchymal cells; after the mesenchymal cells disaggregate *in vitro*, it is intensely expressed in the reaggregated cells [6]. As a transmembrane protein involved in a number of the vital cellular processes, it is an essential participant in the growth and development of the healthy and neoplastic tissues, contributing to the invasive potential of the malignant cells and metastatic spread [1–8]. By interacting with heparin-binding growth factors, it accumulates in the tumor stroma supporting the proliferation and migration of malignant cells, neoangiogenesis, and multiplication of the stroma of invasive tumors [9–13].

The significance of the expression of Sdc1 and its distribution and localization and association with established prognostic factors and prognostic value have been tested so far in pancreatic cancer [14, 15], stomach cancer [16–18], colon cancer [19, 20], liver cancer [21], prostate cancer [22–24], lung cancer [25–28], endometrial cancer [29, 30], ovarian cancer [31], squamous head and neck cancer [32–36], melanoma [37], laryngeal cancer [38], cervical cancer [39], urothelial carcinoma of the bladder [40], multiple myeloma [5, 41–43], and breast cancer [44–46] where its overexpression generally means a poor prognosis, although various studies have shown conflicting results.

The topics that associate the Sdc1 expression in the primary breast tumors and metastases with established prognostic and predictive factors are extremely rare, as well as the significance of the possible associations for the oncology practice. The aim of our study was to determine and compare the immunohistochemical (IHC) expression of Sdc1 in the malignant epithelial cells and tumor stroma of the two, by far, most common histological types of breast cancer, invasive lobular carcinoma (ILC) and invasive ductal carcinoma (IDC), and in the axillary lymph node metastases of ductal type. The goal was also to establish the correlation of the Sdc1 expression with clinical and tumor parameters and the expression of the estrogen/progesterone receptors (ERs/PRs) and HER2/neu oncoprotein in both types of primary tumors and to determine the possibility of defining and isolating the group of tumors with more aggressive biological behavior prone to metastasis.

## 2. Materials and Methods

### 2.1. Materials

We retrospectively analyzed 30 IDC with the axillary lymph node metastasis and 30 ILC archived in the paraffin blocks in the Department of Pathology “Ljudevit Jurak” at the University Hospital Centre “Sestre Milosrdnice” in Zagreb, Croatia, in the period from January 1, 2005, to December 31, 2010. The Sdc1 expression in the lobular cancer metastasis was not taken into consideration because of their lower frequency and lower metastatic potential and therefore an insufficient number of metastatic ILC in the observed period, which we consider to be the limitation of the study. The main clinical and epidemiological data of patients were obtained from the “Thanatos” computer database of the aforementioned Department of Pathology. In order to protect personal information, each tissue sample included in the study was assigned a unique number. The material for analysis was obtained by surgery based on preoperative cytological diagnosis or, rarely, by biopsy of breast tissue. Histopathological diagnosis was made on tumor tissue sections stained by the routine hemalaun-eosin (HE) method. Specimen processing began with prompt standard fixation of tumor tissue in 10 percent buffered formalin, continued by embedding it into paraffin blocks and cutting it in 3–5 *μ*m thick sections, and ended with the HE staining method. All preparations, both histological and IHC, were analyzed by a light microscope Olympus BX41 (Tokyo, Japan). Diagnostic criteria for ductal and lobular carcinomas were based on the mode of growth and the typical architecture of both tumors and on the malignant cell morphology [47]. Considerably simplified tumor-node-metastasis (TNM) classification was used for determining the tumor (T) status and axillary lymph node (N) status [48]. No women at the time of diagnosis had proved distant metastases, and Mx was valid for all.

### 2.2. Immunohistochemical Detection of ERs, PRs, and HER/2neu

All samples were IHC stained to determine the presence of the nuclear ERs and PRs and HER2/neu transmembrane oncoprotein and Sdc1 by using the indirect IHC avidin-biotin complex (ABC) method or the labeled streptavidin-biotin (LSAB) method. The 1D5 monoclonal antibody against the estrogen receptors M7047 (diluted at 1 : 100, Dako, Glostrup, Denmark) and the NCL monoclonal antibody against the progesterone receptors M3569 (diluted at 1 : 100, Novocastra, Newcastle, England) were used in the IHC determination of the ERs and PRs. The results of the immunological reactions were read semiquantitatively in the area showing the strongest staining intensity, the so-called “hot spot.” According to the strength of the IHC reactions or the intensity of nucleus staining, the results were graded as negative (0) (up to 5% of positive tumor cells), weakly positive (1+) (5–10% of positive tumor cells), moderately positive (2+) (10–50% of positive tumor cells), and strongly positive (3+) (over 50% of positive tumor cells).

In IHC determination of HER2/neu, the polyclonal antibody DA485 was used against HER2/neu oncoprotein K5206 (Dako, Glostrup, Denmark). The indirect streptavidin-biotin method was used to visualize the reaction (ChemMate Detection Kit and Peroxidase/DAB in the TechMate device, Dako, Glostrup, Denmark). The staining results were evaluated only in the invasive tumor component, and only the membrane staining was considered positive. The distribution of membrane positivity and the percentage of immunoreactive cells were assessed in the most positive part of the tumor. As in the most laboratories, we used the following evaluation system [49]: 0—no membrane staining or it is present in less than 10% of tumor cells, 1—weak/barely visible membrane staining in more than 10% of tumor cells (partial membrane staining), 2—low/moderate, complete membrane staining in more than 10% of tumor cells, and 3—strong, complete membrane staining in more than 10% of tumor cells.

### 2.3. Immunohistochemical Expression of Sdc1 and “Scoring” System

Sdc1, one of the cluster of differentiation (CD) antigens, categorized as CD138, is mainly expressed on the surface of adherent cells; for example, in adult mice, it is located on the basal and lateral surfaces of the simple epithelia and over the entire surface of the stratified epithelia [50]. During differentiation, stratification, and keratinocyte maturation, when the intercellular adhesion is normally enhanced, the amount of the Sdc1 on the cell surface increases compared to that on the unstratified cells [51, 52], suggesting its direct involvement in the process of the cell adhesion of the contact surfaces. Sdc1 in breast tissue shows the characteristic localization with the IHC intense staining of the basal and lateral surfaces of the normal epithelial cells of most ducts, rarely lobule, while the healthy stromal tissue is not stained [46, 52]. The myoepithelial cells are intensely stained with Sdc1, whereas the luminal cells show heterogeneous reactivity [45]. In the breast cancers, Sdc1 is expressed on the malignant epithelial cells, in the stromal tumor component, and at both locations [46], as also confirmed by our study. The immunoreactivity of tumor cells to the Sdc1 in the invasive carcinomas is different and heterogeneous—it differs from strong diffuse positivity of the tumor epithelium to the focal reactivity of some groups of cells or single cells or to a complete lack of staining (negative reaction). The tumor cells often show the membrane positivity, but sometimes the cytoplasmic one is indicated too. The tumor stroma also shows the heterogeneous Sdc1 expression, so it can be strong, moderate, weak, or absent, and the stromal cells and the collagen fibers can show it too [45, 46]. The dense desmoplastic stroma usually shows strong immunoreactivity, while weaker stromal reaction with stronger lymphoplasmocytic infiltration shows weaker Sdc1 staining [46].

**Figure 1:**
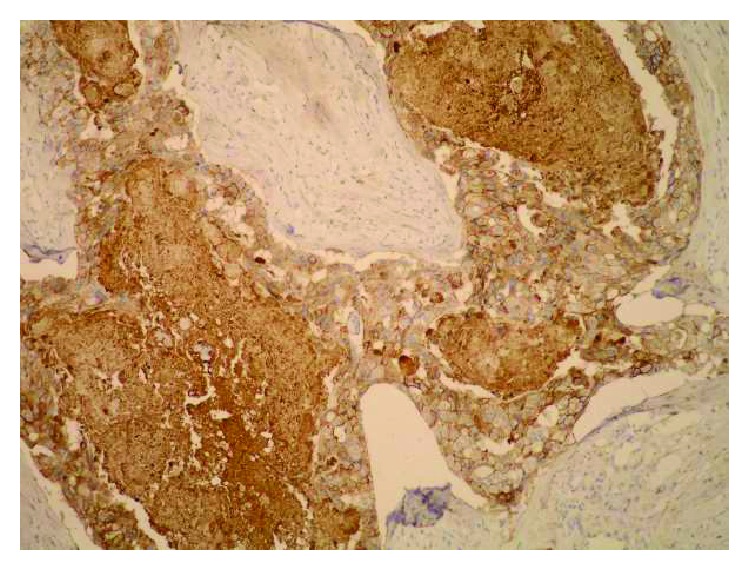
Strong epithelial (both membranous and cytoplasmic) Sdc1 expression and the absence of the stromal Sdc1 expression, with the accumulation of Sdc1 in the intraluminal necrotic tumor mass, IDC (IHC, ×100).

**Figure 2:**
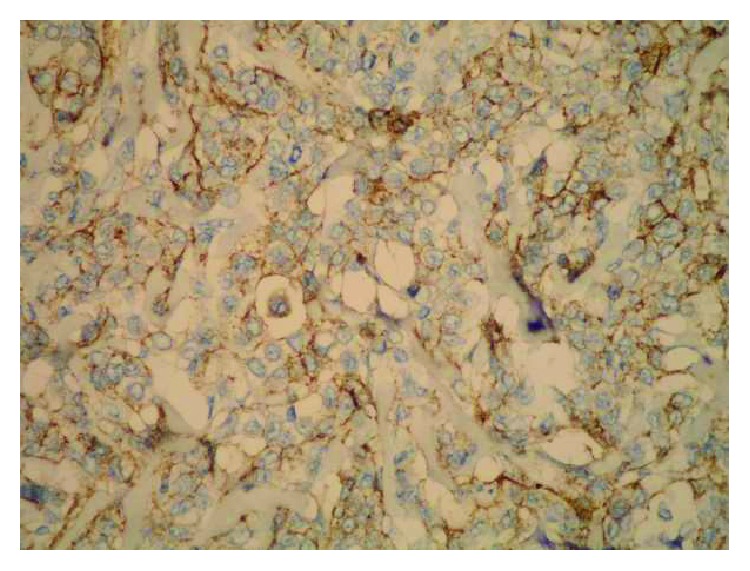
Moderate epithelial Sdc1 expression in the ILC (IHC, ×200).

**Figure 3:**
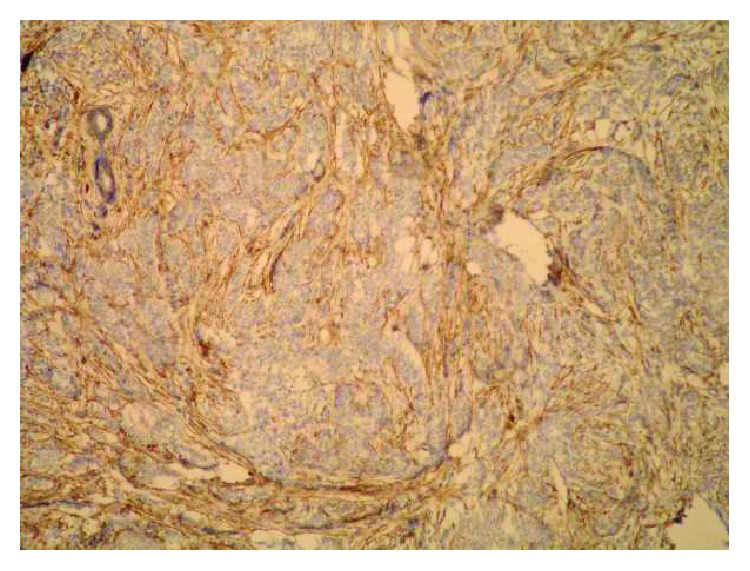
Weak epithelial and strong stromal Sdc1 expression in the ILC (IHC, ×100).

**Figure 4:**
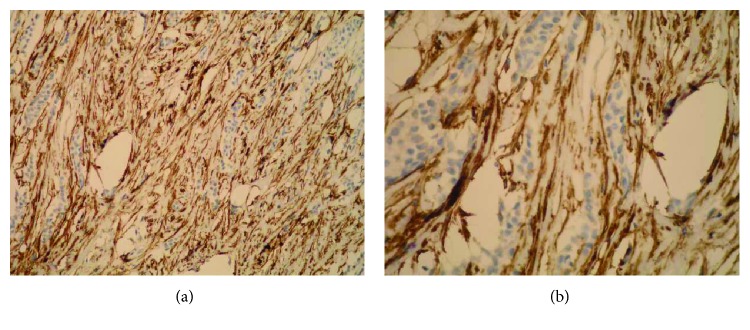
Strong stromal Sdc1 expression in the ILC (the same slide, IHC; ×200 for (a) and ×400 for (b)).

**Figure 5:**
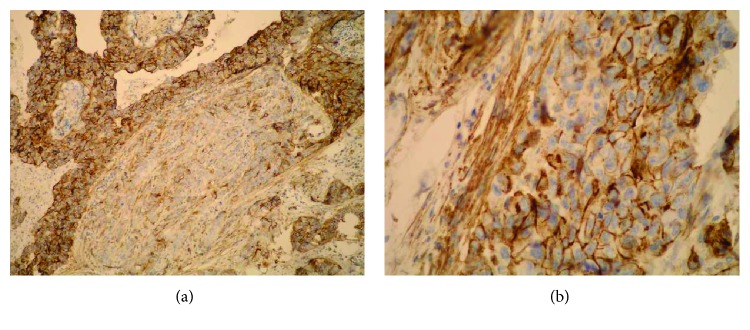
(a) Strong Sdc1 expression in the metastatic epithelium of the ductal carcinoma in the axillary lymph node (IHC, ×100). (b) Strong epithelial (membranous) and stromal Sdc1 expression in the metastasis of the ductal carcinoma in the axillary lymph node (IHC, ×400).

**Figure 6:**
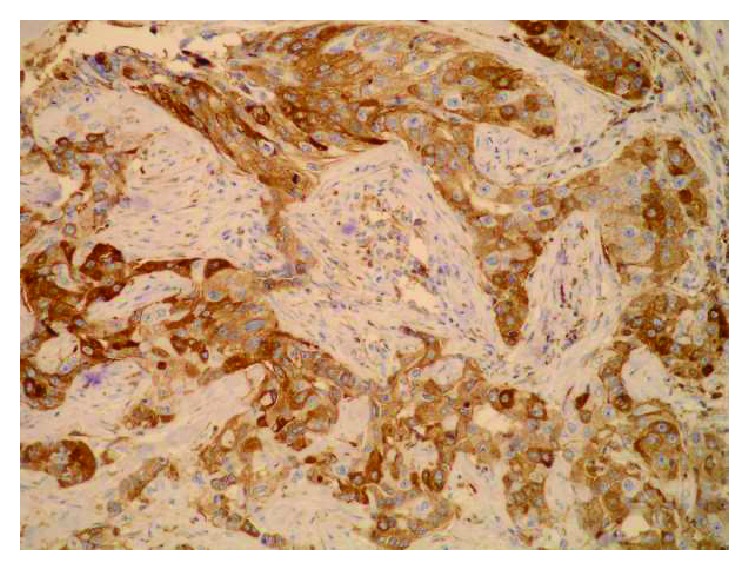
Strong epithelial (both membranous and cytoplasmic) Sdc1 expression in the IDC metastasis in the axillary lymph node (IHC, ×200).

**Figure 7:**
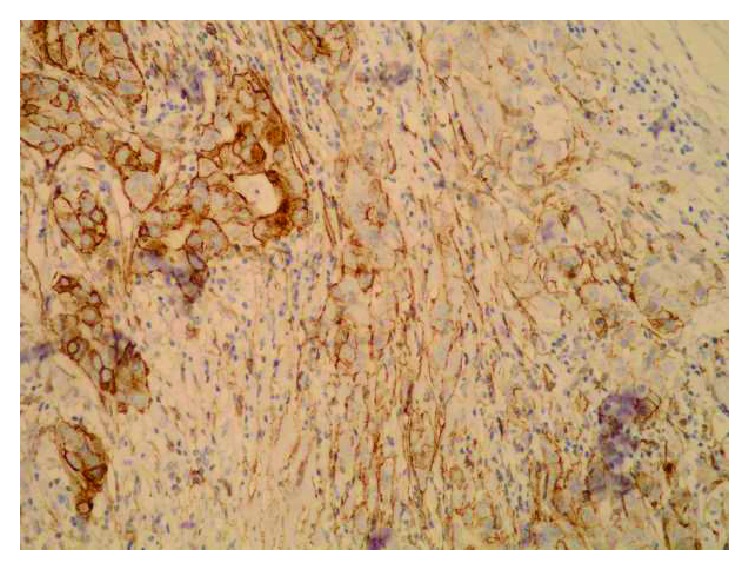
Change of the Sdc1 expression—from the strong epithelial Sdc1 expression to the moderate stromal Sdc1 expression, on the edge of the ductal carcinoma metastasis in the axillary node (subcapsular) (IHC, ×200).

**Figure 8:**
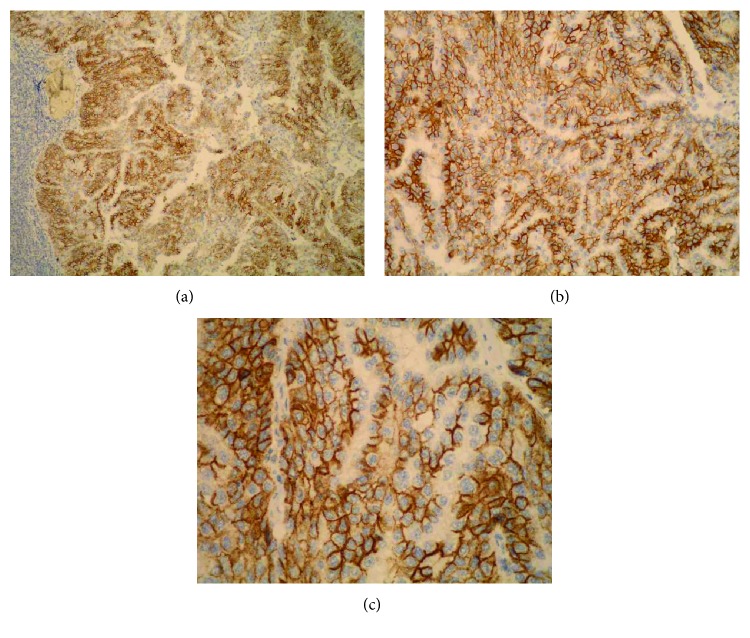
Strong Sdc1 expression in the tumor epithelium of the ductal carcinoma metastasis in the axillary lymph node (the micropapillar histological subtype) (the same slide, IHC; ×100 for (a), ×200 for (b), and ×400 for (c)).

A monoclonal antibody FLEX MO A HU CD138 (diluted at 1 : 50, Dako, Glostrup, Denmark) was used in the study. The indirect ABC technique (LSAB plus kit/HRP, Dako, Glostrup, Denmark) was used for detecting expression. The whole sections of the tumor tissue were examined by two independent examiners (Ivana Miše and Majda Vučić) at the magnification of ×40 and ×100, and then most of the preparation or at least 2000 cells at the magnification of ×400. The proportion of the stained cells and their staining intensity were assessed. Tumors with more than 5% of cells expressing Sdc1 were considered to demonstrate overexpression of Sdc1. The results were expressed by semiquantitative evaluation of the reaction intensity from 0–3 in the area of the greatest intensity of IHC reaction, the strongest staining intensity of the tumor cells (“hot spot”), at an average of five consecutive visual fields at the magnification of ×400 with cca 200 tumor cells [16, 22, 23, 46]. They were graded according to the following: 0 or Sdc1 negative—staining in less than 5% of tumor cells, 1 or weak Sdc1 expression—staining in 5–25% of tumor cells, 2 or moderate Sdc1 expression—staining in 25–50% of tumor cells, and 3 or strong Sdc1 expression—staining in >50% of tumor cells (Figures 1–3). The Sdc1 expression in the stroma of both types of primary tumors, as well as in the tumor epithelium and stroma of the ductal carcinoma metastases in the axillary lymph nodes, was evaluated in the same way (Figures 4–8).

The absence of staining of the normal ductal epithelium or removal of the primary antibody constituted an internal negative control in the preparations of both primary tumors, while the staining of the normal ductal epithelium and/or sometimes the presence of the stratified squamous epithelium of the skin or nipple represented an internal positive control. The normal lymphatic tissue of a lymph node is not stained with Sdc1. The lymphatic tissue of the tonsil constituted the external negative control of the IHC reaction in the axillary lymph nodes, while the positive control was represented by the multilayer squamous epithelium that lines the surface of the tonsil, as well as the plasma cells, located mainly in the medullary cords of the lymph nodes.

### 2.4. Statistical Methods

The results obtained in the study are presented in tables and figures. The chi-square test was used to analyze the differences between categorical variables in relation to the cancer types. The Spearman correlation coefficients between Sdc1 expression in the epithelium and the stroma in both primary and metastatic ductal carcinomas with different clinical and histological parameters were calculated. All *P* values below 0.05 were considered significant. The computer software IBM SPSS Statistics 19.0.0.1 was used to perform the analysis (http://www.ibm.com/analytics/spss-statistics-software, Chicago, IL).

## 3. Results

The clinical and tumor parameters of the primary IDC and ILC included in our research are shown in Table 1.

The statistically significant differences were found between some of the examined parameters of IDC and ILC. They were found in the tumor size (the ductal carcinomas were significantly higher (*P* = 0.002)), in the total number of the isolated lymph nodes (the lobular carcinomas had a significantly greater number of the removed lymph nodes (*P* = 0.006)), in the number of the positive lymph nodes (the ductal carcinomas had a significantly greater number of the positive lymph nodes (*P* = 0.035)), and in the proportion of the positive lymph nodes in relation to the total number of the removed lymph nodes (the ductal carcinomas had a significantly greater number of the positive lymph nodes (*P* = 0.007)) (Table 1).

According to our results, the strong (3+) Sdc1 expression was significantly higher in the tumor epithelium of the primary IDC than in that of the ILC (*P* = 0.027), while there were no significant differences in the stromal expression of Sdc1 between the two types of cancer (*P* = 0.305) (Table 2).

A significant difference in the absence of the Sdc1 expression (null expression) between the tumor epithelium and the stroma of the ductal carcinoma metastases was found (13.3% versus 46.7%, *P* = 0.005) (Table 3). The Yates correction for a small sample also showed a significant difference between them (*P* = 0.011), which means that the stroma of the ductal carcinoma metastases in the axillary lymph nodes more frequently demonstrates the absence of the Sdc1 expression compared to the tumor epithelium of metastases.

The negative correlation between the Sdc1 expression in the tumor epithelium and the PR expression was significant in the ductal carcinomas (*P* = 0.014), as the positive correlation between the Sdc1 expression in the tumor stroma and the histological grade was in the lobular carcinomas (*P* = 0.014) (Table 4). In the total sample of both primary cancers, the positive correlation between the Sdc1 expression in the tumor epithelium with grade (*P* = 0.080) and the expression of the ERs (*P* = 0.068), as well as the positive correlation between the Sdc1 expression in the tumor stroma and the tumor size *P* = 0.063, was marginally significant (Table 4).

The negative correlations between the Sdc1 expression in the malignant epithelium of the metastases with the age of the patients and the ER/PR expression were significant in the ductal carcinoma metastases (*P* = 0.043, *P* = 0.038, and *P* = 0.010) (Table 5). The positive correlation between the Sdc1 expression in the stroma of the metastases and the number of the primary tumor foci (*P* = 0.022) was significant, as well as the negative correlation with the PR expression (*P* = 0.032) in the primary tumors (Table 5).

## 4. Discussion

This study showed that the Sdc1 was expressed in the tumor epithelium in the vast majority or 90% of the primary IDC, and most frequently, it was a strong expression (56.7%). The tumor epithelium of the primary ILC showed the identical overall Sdc1 expression, in 90% of them, but the intensity distribution was slightly different, and it most frequently showed a moderate expression in 56.7% and a strong expression in 26.7% (Table 2). The Chi-square distribution test showed a significantly stronger Sdc1 expression in the primary ductal carcinomas than in the lobular ones (Pearson *χ*^2^ test = 9.17, df = 3, *P* = 0.027). The equal overall epithelial Sdc1 expression in the primary IDC and ILC can be explained by the same origin of both histological types—the epithelium of the terminal ductal lobular unit (TDLU), from which most breast cancers originate [47, 53]. The reason why the molecular profile of the two different histological types of cancer, in spite of the same origin, shows an identical epithelial Sdc1 expression, but, for example, completely opposite E-cadherin expression [47], remains unclear and requires further studies. High levels of Sdc1 are associated with the maintenance of the epithelial cell morphology and the inhibition of invasiveness due to the increased cell adhesion to the matrix components via Sdc1 [54]. The control of the cell adhesion is partly mediated by the Sdc1, and understanding the underlying molecular mechanism, besides the physiological phenomena of growth and development, is necessary in the pathological processes such as tumor cell invasion, angiogenesis, and metastasis [55–57]. By interacting with heparin-binding growth factors, the Sdc1 accumulates in the malignant breast stroma, where its amount can be more than 10 times greater than that in the adjacent normal tissue, with a marked redistribution from the epithelium to the stroma [58], thus contributing to the angiogenesis and proliferation of the stroma of the invasive tumors [59]. In this research, the stroma of the primary IDC demonstrated the overall Sdc1 expression in most tumors (73.3%), with almost equal distribution in all levels (Table 2). The stroma of the ILC included the Sdc1 less frequently, in 60% of tumors. A possible reason for this may be a gentler stromal reaction in the lobular tumors, more pronounced in the elastosis, unlike desmoplasia in the IDC. It is partially weaker due to the typical single-file or “Indian file” arrangement of the tumor cells (in the most common classical histological subtype of the ILC) [47], which makes the contact surface with the surrounding stroma larger. Loussouarn et al. describe a strong immunoreactivity to the Sdc1 of the dense desmoplastic stroma than that of the soft connective stroma [46]. In addition, the change in the Sdc1 expression from the malignant epithelial to the reactive stromal cells [60, 61], with the loss of the E-cadherin, is a critical molecular event in the amazing process of the epithelial-mesenchymal transition (EMT), in which malignant cells lose their epithelial properties and obtain the mesenchymal-like properties in the invasion process [62, 63]. Furthermore, the E-cadherin gene, responsible for the cohesion of the epithelial cells and the suppression of the malignant cell invasion, is absent in 80–100% of the ILC, and its inactivation is an early event in the oncogenesis of the lobular lesions [47]. All the above suggests a stronger connection between the Sdc1 and the E-cadherin in the lobular carcinomas than in the ductal ones. We can assume that the somewhat weaker stromal Sdc1 expression in the lobular tumors is probably associated with a greater E-cadherin loss; that is, the tumor cells of the lobular cancers lose more Sdc1 during the EMT than the ductal carcinoma cells undergoing the EMT. It is supported by the fact that the epithelium of both primary carcinomas expresses the Sdc1 in exactly the same way, in 90% of cases (Table 2). However, either it unevenly loses the Sdc1 during the EMT (which is probably associated with a loss of the E-cadherin as described above) or for some reason, the induction of the Sdc1 is weaker in the stroma of the lobular cancers. Namely, the Sdc1, besides being a transmembrane protein and the native cell membrane HSPG, released from the membrane into the adjacent matrix when excessively expressed [64–66], is partly synthesized in the stroma itself, that is, in the stromal fibroblasts [67–70]. This raises the hypothesis of its possible lower synthesis in the stromal fibroblasts of the ILC, which would be very valuable to investigate. Such a concept is supported by our result of distribution of the Sdc1 expression in the primary ILC (by level), according to which the stroma of the ILC often, in as many as 40%, did not show even a weak expression of the Sdc1 (Table 2). Stanley et al. described a significantly lower epithelial Sdc1 expression in the IDC than in the healthy breast tissue and the epithelial-stromal tumors, with the emphasizing difference in the expression between the stromas of malignant and nonmalignant tissue; the Sdc1 was highly expressed in the stroma and the epithelial-stromal border of the IDC and absent in the stroma of normal tissue and the epithelial-stromal tumors [59]. Such redistribution of the Sdc1 in the direction of the epithelium to the stroma in the malignant epithelial tumors [58] is related to the loss of the Sdc1 expression on/in the malignant epithelial cells [59–61], and it precisely means the EMT—when cancer cells lose their Sdc1 by transition from the epithelial to the weaker differentiated mesenchymal phenotype [62, 63]. The changed or altered Sdc1 expression from the malignant epithelial to the reactive stromal cells is crucial for the transformation of a ductal breast carcinoma to the metastatic disease, but it is also found during progression of other malignant tumors.

In our study, the tumor epithelium of the metastatic IDC in the axillary lymph nodes showed the Sdc1 expression in even 86.7% of metastases, almost the same as the malignant epithelia of the primary IDC; the Sdc1 expression was mostly strong (50%) or moderate (30%), while 13.3% of the metastatic tumor epithelium did not show even a weak expression (Table 3). Thanakit et al. also found a high expression of the Sdc1 in the affected axillary nodes at the primary IDC, with a significant expression increase during the tumor progression from the lymph node to the extracapsular adipose tissue [71], while the E-cadherin expression showed no significant difference between the metastatic nodes and the extracapsular tumor invasion. According to our results, a high Sdc1 expression in the tumor epithelium of the metastasis, almost equal to that of a primary tumor, suggests direct involvement of the Sdc1 in progression, not only of primary ductal carcinomas but also of metastatic carcinomas. It is very important to emphasize that this strong Sdc1 expression in the tumor epithelium of lymph nodes might be a possible prerequisite for the further progression and metastasis of a ductal carcinoma, in the loco-regional and remote areas. However, Wang et al. found a significantly reduced Sdc1 expression in the metastatic breast cancer cell lines compared to the nonmetastatic breast cancer cell lines under *in vitro* conditions [72]. Our study determined that the stroma of the metastatic IDC showed an overall Sdc1 expression in 53.4% of the lymph nodes (i.e., it was absent in almost half of them or 46.7% (Table 3)), which is lower (but not statistically significant) than the expression in the stroma of the primary ductal carcinomas (73.3%). The probable reason for this is the smaller amount of stromas in a lymph node and therefore its lower reactivity to the metastatic epithelium compared to the primary tumors (a lymph node shows a discrete stroma in the physiological state too), and also a different microenvironment for the metastatic epithelium in the new “host”—the lymph node, which determines different epithelial-stromal interactions.

The HER2/neu is overexpressed in 25–30% of the invasive breast cancers and even in 50% of the breast ductal carcinoma in situ (DCIS) [49]. Götte et al. demonstrated a strong Sdc1 expression in 72% of the DCIS, with a correlation between the levels of Sdc1 and HER2/neu expression [73]. Kim et al. showed a significant association between the epithelial Sdc1 and EGFR expression in the colorectal carcinomas [74]. It seems that some syndecans (Sdc1 and Sdc4) play a key role in the activation of the *α*6*β*4 integrin by receptor tyrosine kinases (HER1 and HER2) [75]. Besides, the action of a trastuzumab, an antibody in the therapy of the breast cancer cases positive for human EGFR2 (HER2/neu), depends on the availability of the heparan sulfate on the surface of the breast cancer cell lines [76]. All these indicate an association between the Sdc1 and HER2/neu, that is, their signaling pathways. Nevertheless, our total sample of the primary tumors did not demonstrate it, most likely because the half of them were the lobular carcinomas in which the HER2/neu is rarely expressed [47, 77–79], nor the IDC, probably because of the weaker HER2/neu expression in the invasive carcinomas than in in situ carcinomas [49]. Since HER2/neu is particularly expressed in the carcinomas in situ, it is probably more involved in the initiation of carcinogenesis than in the growth of the already established tumors, hence showing no correlation with the Sdc1 in our research.

In this study, the lobular carcinomas demonstrated a positive correlation between the stromal Sdc1 expression and histological grade (*P* = 0.014) (Table 4). Loussouarn et al. associated a strong epithelial Sdc1 expression with a low-grade and well-differentiated breast carcinomas and a reduction of expression with poorly differentiated ones [46]. Leivonen et al. discovered an epithelial Sdc1 expression in 61% of the IDC [44], which is lower than our result, and a stromal expression in 67%, which is very similar to our result. Barbareschi et al. identified the increased Sdc1 expression in 42% of cancers, mostly in large tumors, with a high grade and a high mitotic index, a negative ER/PR status, and an HER2/neu overexpression [45]. According to our results, the primary IDC showed a significant negative correlation between the epithelial Sdc1 expression and the PR expression (*P* = 0.014) (Table 4). The axillary node metastases from the IDC demonstrated a negative relationship between the Sdc1 expressed in the tumor epithelium and the patient age (*P* = 0.043), as well as the ER (*P* = 0.038) and PR expression (*P* = 0.010) in the primary tumors (Table 5). Thus, a higher expression of the Sdc1 in the tumor epithelium of the metastatic IDC is associated with younger patients and a lower expression of both hormone receptors in the primary tumors, which is very significant and can be a basis for further studies. The Sdc1 expression in the stroma of metastases positively correlated with the number of tumor foci (*P* = 0.022) in the primary tumor and negatively correlated with the PR expression (*P* = 0.032) in the primary tumor. Baba et al. related the Sdc1 overexpression (but in the primary tumor) and the negative ERs to the aggressive, highly proliferative type of a breast cancer [80]. Leivonen et al. associated the epithelial Sdc1 expression with the negative ERs and the stromal Sdc1 expression with the positive ERs [44]. As noted, Barbareschi et al. also have linked the Sdc1 expression (in a primary tumor) with the negative ER/PR [45]. All the above indicates the existence of certain relations between the Sdc1 expression and ER/PR status in the primary ductal carcinomas, while the results trying to define this relationship in the metastases were not found in the reviewed literature.

## 5. Conclusion

The aim of our study was to determine and compare the Sdc1 expression in the malignant epithelial cells and stroma of 30 ILCs and 30 IDCs, as well as in the axillary lymph node metastases of ductal type, and to correlate it with the clinical and tumor parameters. This research has shown the identical overall epithelial Sdc1 expression with no statistically significant difference in its stromal expression between by far the two most common primary breast cancers—ductal and lobular cancers. However, it has shown some differences in the correlation between the Sdc1 expression and the important hormonal ER/PR status as the unavoidable prognostic/predictive factors in the routine diagnostic-therapeutic procedure of each breast carcinoma. The involvement of Sdc1 in the progression of both primary cancers was proved, as well as the involvement of Sdc1 in the development of the metastatic potential of ductal tumors when invading the axillary lymph nodes. Moreover, the frequent and strong Sdc1 expression in the nodal metastasis (found in almost 90% of cases) assumes a very high probability of further disintegration of the malignant cells, and it presents a significant source of the new metastases. A further research on a larger number of patients with different types of breast cancer is needed in order to define the role and behavior of the Sdc1 in different histologic tumor types and to include the results of selected types of the Sdc1 expression (both are positive/negative or one of them is positive) into the comprehensive molecular and gene profile at the level of an individual tumor. Such research will continue the path towards understanding the numerous mutually dependent or autonomous molecular processes in the complex biopathology and carcinogenesis of breast cancer.

## Figures and Tables

**Table 1:** Differences in the investigated quantitative values between the invasive ductal and lobular breast carcinomas: the statistical analysis was done with the Mann–Whitney *U* test.

	Histological types	*N*	Arithmetic mean	Standard deviation	Min	Max	Percentiles			*P* value
							25	Median	75	
Age (years)	Ductal carcinoma	30	65.13	14.98	37	87	55.00	**67.00**	77.75	0.096
	Lobular carcinoma	30	59.63	11.99	36	77	50.75	**63.50**	69.50	

Tumor size (cm)	Ductal carcinoma	30	3.89	1.59	1.20	8.50	2.50	**3.65**	5.05	**0.002**
	Lobular carcinoma	30	2.77	1.90	0.70	8.00	1.50	**2.00**	3.28	

Total number of lymph nodes	Ductal carcinoma	30	10.80	4.48	4	21	6.75	**11.00**	14.00	**0.006**
	Lobular carcinoma	29^∗^	14.45	4.59	8	26	10.50	**13.00**	17.00	

Number of positive lymph nodes	Ductal carcinoma	30	5.27	3.48	1	13	2.00	**4.00**	8.00	**0.035**
	Lobular carcinoma	29^∗^	4.90	7.26	0	25	0.00	**2.00**	6.00	

Percentage of positive lymph nodes (%)	Ductal carcinoma	30	50.92	28.66	5.26	100.00	26.70	**46.43**	77.00	**0.007**
	Lobular carcinoma	29^∗^	30.58	36.72	0.00	100.00	0.00	**11.76**	53.57	

^∗^For one invasive lobular carcinoma, the number of the total isolated and positive lymph nodes was not known (Nx).

**Table 2:** The distribution of the intensity of the Sdc1 expression by the levels in the tumor epithelium and the stroma of primary IDC and ILC.

		Histological types				Chi-square value	*P*
		Ductal carcinoma		Lobular carcinoma			
		*N*	%	*N*	%		
Syndecan-1 expression in the tumor epithelium	0	3	10.0	3	10.0	9.17	**0.027**
	1	4	13.3	2	6.7		
	2	6	20.0	17	56.7		
	3	17	56.7	8	26.7		

Syndecan-1 expression in the tumor stroma	0	8	26.7	12	40.0	3.62	0.305
	1	8	26.7	5	16.7		
	2	7	23.3	3	10.0		
	3	7	23.3	10	33.3		

**Table 3:** The distribution of the intensity of the Sdc1 expression by levels in the tumor epithelium and the stroma of the metastases of the ductal breast cancer in the axillary nodes.

Metastases of ductal carcinoma		Intensity of syndecan-1expression				Total
		0	1	2	3	
Tumor epithelium of metastases	*N*	4	2	9	15	30
	%	13.3	6.7	30.0	50.0	100.0

Stroma of metastases	*N*	14	5	5	6	30
	%	46.7	16.7	16.7	20.0	100.0

**Table 4:** The correlation between the Sdc1 expression in the tumor epithelium and the stroma of the total sample of both types of the primary tumors and separately of the invasive ductal and the lobular carcinomas, with various clinical and histological parameters analyzed using Spearman's correlation coefficient.

Spearman correlation		Syndecan-1 expression in the tumor epithelium			Syndecan-1 expression in the tumor stroma		
		Total sample (*N* = 60)	Ductal carcinoma (*N* = 30)	Lobular carcinoma (*N* = 30)	Total sample (*N* = 60	Ductal carcinoma (*N* = 30)	Lobular carcinoma (*N* = 30)
Age (years)	Coefficient rho	−0.159	−0.179	−0.288	−0.1	0.096	−0.346
	*P*	0.225	0.345	0.123	0.447	0.612	0.061

Tumor size (cm)	Coefficient rho	0.141	0.129	−0.021	0.242	0.114	0.28
	*P*	0.283	0.498	0.912	0.063	0.548	0.134

Focus numbers (1–3)	Coefficient rho	0.103	−0.007	0.295	−0.099	−0.139	−0.06
	*P*	0.435	0.97	0.113	0.451	0.463	0.752

Histological grade	Coefficient rho	0.228	0.185	0.078	0.187	0.000	0.442
	*P*	0.08	0.329	0.683	0.153	0.999	**0.014**

T status	Coefficient rho	0.023	−0.253	0.025	0.203	0.145	0.254
	*P*	0.863	0.177	0.895	0.119	0.443	0.175

N status	Coefficient rho	0.176	0.136	0.115	0.122	0.062	0.169
	*P*	0.182	0.475	0.553	0.357	0.744	0.38

Percentage of positive lymph nodes	Coefficient rho	0.213	0.254	0.071	0.002	−0.153	0.11
	*P*	0.105	0.175	0.713	0.989	0.421	0.57

ER	Coefficient rho	−0.237	−0.205	−0.268	−0.034	−0.099	0.021
	*P*	0.068	0.277	0.152	0.799	0.604	0.911

PR	Coefficient rho	−0.156	−0.442	0.187	0.022	−0.007	0.034
	*P*	0.235	**0.014**	0.323	0.868	0.971	0.86

HER2/neu	Coefficient rho	−0.054	−0.09	−0.052	−0.028	−0.101	0.072
	*P*	0.683	0.637	0.787	0.831	0.597	0.705

**Table 5:** The correlation between the Sdc1 expression in the tumor epithelium and the stroma of the metastases of the ductal breast carcinoma and some clinical and histological features of the primary tumors, analyzed using Spearman's correlation coefficient.

Spearman correlation		Syndecan-1 expression in the epithelium of metastases (*N* = 30)	Syndecan-1 expression in the stroma of metastases (*N* = 30)
Age (years)	Coefficient rho	−0.373	0.014
	*P*	**0.043**	0.940

Tumor size (cm)	Coefficient rho	−0.223	0.061
	*P*	0.237	0.750

Focus numbers (1–3)	Coefficient rho	0.07	0.417
	*P*	0.714	**0.022**

Histological grade	Coefficient rho	0.138	0.164
	*P*	0.466	0.385

T status	Coefficient rho	−0.299	0.229
	*P*	0.109	0.223

N status	Coefficient rho	0.11	0.179
	*P*	0.562	0.343

Percentage of positive lymph nodes	Coefficient rho	0.08	0.267
	*P*	0.676	0.153

ER	Coefficient rho	−0.381	−0.302
	*P*	**0.038**	0.105

PR	Coefficient rho	−0.461	−0.393
	*P*	**0.01**	**0.032**

HER2/neu	Coefficient rho	0.138	0.287
	*P*	0.468	0.124

## Data Availability

The data that support the findings of this study are available from the corresponding author upon reasonable request.
